# Shortening duration of ertapenem in outpatient parenteral antimicrobial therapy for complicated urinary tract infections: A retrospective study

**DOI:** 10.1371/journal.pone.0223130

**Published:** 2019-09-26

**Authors:** Doug L. Fink, Steven Collins, Ronnie Barret, Gabriele Pollara, Michael Marks, Sarah Logan

**Affiliations:** 1 Department of Microbiology, University College London Hospital, London, United Kingdom; 2 Division of Infection and Immunity, University College London, London, United Kingdom; 3 Hospital for Tropical Diseases, University College London Hospital, London, United Kingdom; 4 Clinical Research Department, Faculty of Infectious and Tropical Diseases, London School of Hygiene & Tropical Medicine Hospital for Tropical Diseases, University College London Hospitals NHS Trust, London, United Kingdom; Instituto Nacional de Ciencias Medicas y Nutricion Salvador Zubiran, MEXICO

## Abstract

**Background:**

The incidence of multi-drug resistant ESBL-associated urinary tract infections (UTIs) is increasing globally. Patients with abnormal renal tract anatomy and other co-morbidities are at increased risk of complicated UTI and ESBL-associated infections. The duration and safety of OPAT for this cohort of patients is unknown.

**Objectives:**

This study aims to provide an evidence base to support decision-making regarding duration of antibiotic treatment for complicated UTIs.

**Methods:**

We retrospectively reviewed all patients receiving ertapenem with or without adjunctive fosfomycin for complicated UTIs in the OPAT service of our tertiary infectious diseases hospital. All data had been collected prospectively as part of routine clinical care. Our primary outcomes were microbiological and clinical cure of UTI.

**Results:**

We identified 33 treatment episodes of ertapenem use for UTIs. 76% episodes related to pyelonephritis or urosepsis diagnoses. Renal tract abnormalities or prior urological surgery were present in 45% of patients. The median duration of appropriate parenteral antibiotic therapy in our study was 6 days. Clinical cure was achieved with short-course parenteral treatment alone in 81% of patients and this increased to 96% when adjunctive fosfomycin was used. There was a single treatment failure resulting in hospital admission.

**Conclusions:**

Short duration ertapenem via OPAT with or without adjunctive fosfomycin is safe and effective for the treatment of complicated UTIs. Further studies are required to inform optimal treatment strategies and publication of guidelines in this field.

## Introduction

Antimicrobial resistance (AMR) is associated with increased morbidity, mortality and prolonged hospital admission [1, 2]. A major challenge for contemporary health services is the provision of antimicrobial therapy against resistant organisms that is effective, acceptable to patients and cost-effective. Outpatient parenteral antibiotic therapy (OPAT) facilitates the administration of injectable agents to patients outside of hospital who would historically have required prolonged hospital admission. Both patients and health care providers may benefit from shortening in-hospital stays and transferring care into a community or outpatient setting [3].

There is an increasing incidence of community- and hospital-acquired multi-drug resistant extended spectrum beta-lactamase (ESBL)-associated urinary tracts infections (UTIs) [4]. Carbapenems are often used as first line therapy for individuals requiring injectable agents [5]. Once daily ertapenem is emerging as a pragmatic and well-tolerated choice for OPAT in this setting if there is no other indication for hospital admission [6]. Alongside carbapenems there is growing use of older-agents such as fosfomycin for the treatment of ESBL UTIs [7]. Currently UK marketing authorisation of fosfomycin is restricted to oral therapy for acute lower uncomplicated UTIs but there is increasing interest in using fosfomycin as an oral adjunct or an alternative intravenous drug for the treatment of complicated UTIs including those associated with bacteraemia [8, 9]. There are very limited data regarding oral fosfomycin use for this latter group in the UK and no prospective studies [10].

There remains a paucity of high-quality randomized control trial (RCT) data to guide duration of antimicrobial therapy for upper tract UTIs. European guidelines advocate up to 21 days of antimicrobial therapy for complicated UTIs [11]. In the setting of uncomplicated UTIs, meta-analyses have published conflicting findings regarding efficacy of short course therapy lasting less than 14 days compared with long courses lasting 14 days or more [12, 13]. Shortening antibiotic treatments may have significant benefits to antimicrobial stewardship, cost and patient experience if it can be achieved without compromising clinical care.

History of urological surgery is an independent risk factor for ESBL-associated UTIs [14] (. This may be associated with an increasingly elderly patient population with multiple co-morbidities undergoing ever-more complex surgery [15, 16]. Surgical patients with complicated UTIs are underrepresented in the existing OPAT literature [6, 17].

In this study we retrospectively reviewed the clinical and microbiological outcomes of a prospectively enrolled cohort of patients receiving ertapenem with or without adjunctive fosfomycin via OPAT for UTI.

## Materials and methods

The study included patients managed through the OPAT service at University College London Hospitals (UCLH), a tertiary referral centre in London, United Kingdom. At UCLH patients are accepted onto the OPAT service after clinical review by at least one infection consultant on any day of the week. Consequently, all OPAT patients are clinically reviewed and outcomes determined weekly by a multidisciplinary team, including at least two infection specialists. Treatment decisions including selection of appropriate antimicrobials, length of treatment and addition of oral therapy following OPAT are made by the OPAT MDT. Provision of treatment is most frequently by nursing staff in the patient’s own home although occasionally patients come to UCLH itself for daily administration of OPAT.

### Data collection

We included any patient who completed a course of ertapenem with or without fosfomycin through OPAT for a UTI over a 16-month period between January 2015 and August 2016. Demographic, clinical and microbiological data were collected prospectively into an electronic database used to manage all patients seen by the infectious diseases inpatient and OPAT service [18].

### Diagnostic investigations

A urine sample was collected from all patients as part of routine clinical management. Analysis included the presence or absence of leucocytes and nitrites. Bacterial colonies grown on solid agar plates were identified by MALDI-TOF. Antibiotic susceptibility testing was performed by the disc diffusion method and Vitek in line with EUCAST recommendations. Blood cultures and renal tract imaging were collected at the discretion of the clinical team.

### Treatment and outcome definitions

Urinary tract infections were classified as complicated or uncomplicated in line with the European Urology Association (EUA) Urological Infection guidelines [11]. Complicated infections were defined as any infection associated with a risk factor that increased the likelihood of treatment failure. For the purposes of this study we did not consider AMR alone as defining a complicated UTI.

We defined 14 days as standard antibiotic treatment duration for pyelonephritis and acute bacterial prostatitis, and 28 days for chronic bacterial prostatitis as suggested by EUA guidelines to calculate days of treatment saved. This is an intentionally conservative definition. We did not include patients diagnosed with urosepsis defined by EUA guidelines in the calculation of days of treatment saved, as treatment guidelines for this diagnosis recognise the heterogeneity of such patients and do not mandate treatment duration. Adjunctive fosfomycin is defined as fosfomycin therapy following initial ertapenem treatment and was considered when the patient had not achieved clinical cure at the end of their course of ertapenem. Inappropriate antibiotic use was defined as use of agents to which isolated organisms were proven to be resistant on culture taken at the start of or during the episode of antibiotic therapy. Ertapenem and Fosfomycin were both prescribed at a standard dose of 1g once a day intravenously and 3g orally once every three days respectively.

Microbiological cure was defined as a negative culture on any occasion after treatment. Clinical cure was defined as complete resolution of symptoms and signs of UTI. Treatment episodes characterised by adverse drug reactions in addition to change of therapy, hospital re-admission due to UTI or mortality were considered treatment failures.

### Ethics

The study was reviewed and approved by the Audit and Research Committee at the Hospital for Tropical Diseases, University College Hospital. The stated that as this was a retrospective case note review, meeting the NHS definition of service evaluation, formal ethical approval was not required.

## Results

### OPAT referrals

A total of 30 patients received 33 treatment courses of ertapenem during the period of the study. The mean age of the cohort was 49 years (range 22–91) and 43% of patients were male. Acute pyelonephritis was the most common OPAT referral indication (57.6%). Almost half of the cohort had abnormal renal tracts or prior urological surgery (45%) and one third had risk factors for a complicated UTI as defined by EUA guidelines. Almost all patients were referred from inpatient services (82%), most commonly infectious diseases (50%) and urology (27%) (**Table 1**)

**Table 1:** Clinical and microbiological characteristics of patients.

**Age in years** **(Mean and Range)**		49 (22–91)
**Male**		13 (43%)
**Indication**	Pyelonephritis	19 (57.6%)
	Urosepsis	6 (18.2%)
	Urinary Tract Infection	5 (15.2%)
	Prostatitis	3 (9.0%)
**Microbiology**	ESBL *E*.*coli*	20 (60.6%)
	Other ESBL	2 (6.1%)
	AmpC Producer	2 (6.1%)
	Other	2 (6.1%)
	No positive sample*	7 (21.1%)
**Microbiology for patients receiving OPAT ertapenem**	ESBL/AmpC Producer	31 (93%)
	Drug Allergy	2 (7%)

* All patients without a positive microbiology sample during the OPAT episode of care had a previous ESBL isolate within the previous 12-month period.

### Microbiology and investigations

Bacteria were isolated in 79% (26/33) of treatment episodes. Of these, 21 patients had a positive mid-stream urine and 5 patients had a positive blood culture. All patients without positive samples during the index treatment episode had urinary specimens in the preceding 12 months that had grown ESBL-producing isolates. Bacteria were identified from blood culture in 5 patients (5/33, 15%). ESBL-producing organisms accounted for 92% of isolates (24/26), with *Escherichia coli* being the most common ESBL producer (83%). *Klebsiella pneumoniae* was the next most common organism (n = 3, 12%). Two patients with non-ESBL-producing UTIs required parenteral ertapenem therapy in the absence of oral therapeutic options owing to drug allergy. Of the ESBL-producing isolates 83% (20/24) were sensitive to fosfomycin **(Table 1)**. Renal tract imaging was performed in 66% of patients (20/33).

### Treatment

In 12 (12/33, 36%) cases, isolated organisms were resistant to initial antimicrobial therapy, most commonly cefuroxime, the first-line antimicrobial for upper-tract UTIs at UCLH. The mean duration of inappropriate antibiotic use was 3 days prior to switching (range 1 to 5 days). The median time from initial treatment to OPAT referral was 3 days (IQR 1–4 days). For patients diagnosed with pyelonephritis or urosepsis the median duration of appropriate injectable antibiotic therapy was 6 days (range 3 to 15 days). 72% of this group had complicated UTIs (18/33). The mean duration of OPAT ertapenem in this group was 6 days (range 2 to 9 days). For 63% of treatment episodes (21/33) all antibiotics were delivered through peripheral cannulae alone. Adjunctive fosfomycin was used following ertapenem for 6 treatment episodes (18.2%) 117 treatment days were saved in 22 individuals with upper tract UTIs (excluding urosepsis) compared with the duration of therapy had standard treatment courses in line with European guidelines been prescribed.

### Outcomes

All patients were reviewed within 7 days of completing OPAT. Clinical cure was seen in 81% of treatment episodes (27/33) following treatment with ertapenem alone. All patients receiving adjunctive fosfomycin achieved clinical cure, and therefore including these patients, the overall cure rate was 97% (32/33 patients). There was one treatment failure where a patient required readmission to hospital with urosepsis. Two intravenous catheter extravasation injuries occurred during OPAT resulting in change of intravenous catheters. There were no recorded adverse drug reactions and no episodes of *Clostridium difficile*.

## Discussion

In this study we provide data to support the use of short courses of ertapenem and adjunctive fosfomycin delivered though an OPAT service for the treatment of ESBL-associated complicated UTIs. The mean duration of appropriate injectable antimicrobial therapy for the diagnoses of pyelonephritis or urosepsis was 7 days. Overall, we were able to successfully limit treatment duration to 7 days in 81% of patients and achieve clinical cure overall in 96% of patients. The duration of treatment in this study is significantly shorter than the minimum duration of antimicrobial therapy suggested by international guidelines for complicated UTIs and that delivered in other cohort studies [6, 11, 17] and suggests that treatment duration may be shortened whilst maintaining clinical outcomes. Besides its efficacy and tolerability, the shorter courses of parenteral therapy delivered in our study reduced the need for long line indwelling intravascular catheters which are associated with an increased rate of complications [19].

The current UK licence for fosfomycin is limited to acute lower uncomplicated UTIs. In this study adjunctive fosfomycin increases the clinical cure rate of complex urinary tract infections. Whether fosfomycin could be used as an initial or single carbapenem sparing treatment for complex ESBL UTIs requires further study [9].

The main limitation of the study is the relatively small size of the cohort studied and the non-randomised nature of the study. The majority of patients had 2 or more risk factors for complicated UTIs and 45% had abnormal renal tracts, although this was performed at the discretion of clinicians, possibly introducing bias in our cohort. Nevertheless, complicated UTIs constitute a growing proportion of OPAT cases but are often excluded from trials, and therefore our data add to the efficacy and safety evidence for OPAT ertapenem and fosfomycin to treat urological infections.

Duration of treatment is a balance between efficacy and tolerability. A formal randomised trial to compare longer and shorter durations of antibiotics in this setting should be considered to provide definitive data on optimal treatment duration [20]. Additional studies on the use of fosfomycin in this setting are warranted. Our data suggest that short courses of parenteral ertapenem with or without adjunctive fosfomycin delivered via OPAT is an effective, safe and cost-effective strategy for managing these infections.

## Supporting information

S1 Data Supplementary dataset. (XLS)
